# The Alternatives to Traditional Materials for Subsoil Stabilization and Embankments

**DOI:** 10.3390/ma12183018

**Published:** 2019-09-18

**Authors:** Mirjana Vukićević, Miloš Marjanović, Veljko Pujević, Sanja Jocković

**Affiliations:** Faculty of Civil Engineering, University of Belgrade, Bulevar kralja Aleksandra 73, 11000 Belgrade, Serbia; mirav@grf.bg.ac.rs (M.V.); vpujevic@grf.bg.ac.rs (V.P.); borovina@grf.bg.ac.rs (S.J.)

**Keywords:** fly ash, slag, soil stabilization, embankment, cement, lime

## Abstract

Major infrastructure projects require significant amount of natural materials, often followed by the soft soil stabilization using hydraulic binders. This paper presents the results of a laboratory study of alternative waste materials (fly ash and slag) that can be used for earthworks. Results of high plasticity clay stabilization using fly ash from Serbian power plants are presented in the first part. In the second part of the paper, engineering properties of ash and ash-slag mixtures are discussed with the emphasis on the application in road subgrade and embankment construction. Physical and mechanical properties were determined via following laboratory tests: Specific gravity, grain size distribution, the moisture–density relationship (Proctor compaction test), unconfined compressive strength (UCS), oedometer and swell tests, direct shear and the California bearing ratio (CBR). The results indicate the positive effects of the clay stabilization using fly ash, in terms of increasing strength and stiffness and reducing expansivity. Fly ashes and ash-slag mixtures have also comparable mechanical properties with sands, which in combination with multiple other benefits (lower energy consumption and CO_2_ emission, saving of natural materials and smaller waste landfill areas), make them suitable fill materials for embankments, especially considering the necessity for sustainable development.

## 1. Introduction

The modern world is facing the consequences of the technological development followed by a huge environmental impact. This has stimulated recent scientific research in the field of identifying pollutants and the possibility of reducing the harmful effects of pollution. One of the major pollutants is fossil fuel, which produce huge amounts of CO_2_ in the combustion process. According to the World Bank data for 2015 [1], the total share of fossil fuels in energy production in the world is 65.2%. According to the same data in Serbia, this share is 73.1%, since the thermal power plants are the main producers of electricity. There are six thermal power plants within the Electric Power Industry of Serbia, which use lignite as the main fuel.

Thermal power plants have multiple harmful effects on the environment: They pollute air by emitting CO_2_, SO_2_, N_2_O and fly ash; landfills of ash and slag occupy large areas of mainly agriculture land; deposited ash can potentially pollute land and water due to the presence of microelements and radionuclides. The harmful effects of flue gases can be reduced by gas desulphurization, installation of efficient electro filters and application of methods for reducing the concentration of nitrogen oxides. The amount of deposited ash and slag can be significantly reduced by use in the construction industry.

According to the ECOBA (European Coal Combustion Products Association) data for 2016 [2], annual production of ash in the European Union (EU 15) was about 40 million tons, of which 64% was fly ash. About 50% of the produced amount was used in the construction industry, 41.5% was used for land reclamation–restoration and only 6.7% was deposited. In the construction industry, it was mostly used for the production of cement and concrete (about 25%), while much less (about 6% today vs. 25% ten years ago) was used in the road construction. The data show that ash in developed EU countries is successfully used as raw material in the industry. In Serbia, the situation is completely different. About 7 million tons of ash is produced annually. A very small part of the ash is deposited in silos, while most of the total produced amount is deposited with the slag at the landfills. The landfills occupy an area of about 1600 ha, with about 300 million tons of ash and slag [3,4]. So far, only 3% of ash has been used for the production of cement [4].

In Serbia, the first major research related to the possibility of using ash in road construction began in the first decade of this century, with the aim of reducing the large amount of deposited ash. Since then, four extensive studies have been done [5,6,7,8]. Based on the results of these studies, in 2015 the Serbian Government has passed a regulation [9] on the use of ash from thermal power plants in Serbia, thus creating a legal framework for the use of ash.

During the research [7,8], about 1000 laboratory tests of physical and mechanical properties of fly ash, ash and slag, mixtures of ash and soil with or without hydraulic binders (cement and lime) were done in the Laboratory for Soil Mechanics of the Faculty of Civil Engineering in Belgrade. Additionally, chemical composition of fly ash was investigated. The main results and conclusions from these studies are presented in this paper.

The aforementioned studies included a very important ecological aspect of the use of ash, bearing in mind that ash contains harmful substances that can be a potential source of pollution of soil and water. Ash can be disposed of as waste material if the content of artificial and natural radionuclides is less than the values prescribed in the Rulebook on the Limits of Radioactive Contamination of the Environment [10]. Ash and slag from the Serbian thermal power plants meet the prescribed requirements [11]. Ash also contains trace elements that are hazardous to the environment, such as As, B, Cr, Mo and Se [12]. These elements could contaminate the soil, water and marine ecosystems in case of their leaching. The main factor in the control of leaching is the control of the mobility of the trace elements. There are appropriate procedures that can reduce or eliminate the leaching of toxic trace elements such as As, B, Cr, Sb and Se [11,12]. If it is proven that there is no risk of leaching, the use of ash for embankments provides economic and environmental benefits.

In the first part of the paper, the results of high plasticity clay stabilization using fly ash from Serbian power plants (with and without binders) were presented. In addition, the effects of ash application as a soil stabilizer were compared with the effects of chemical additives for the same purpose. In the second part, engineering properties of ash and ash-slag mixtures as embankment material in road construction were investigated. The mechanical properties important for fulfillment of the technical requirements for road subgrade were tested. The influence of common binders (activators) was also investigated.

## 2. Literature Review

### 2.1. Ash Utilization for Soil Stabilization

The factors on which soil stabilization with ash depend on are: The type of ash and its characteristics, characteristics of the soil to be stabilized, the percentage of fly ash, the time period between wetting of the mixture and compaction and soil water content at the time of compaction. 

According to ASTM C618 [13], fly ash types are class C and class F. This classification mainly depends on the content of SiO_2_, Al_2_O_3_ and Fe_2_O_3_—minimum percentage of SiO_2_ + Al_2_O_3_ + Fe_2_O_3_ for class F fly ash is 70%, and for class C is 50%. The percentage of sulfur trioxide (SO_3_) is max 5% for both ash classes (fly ash with a sulfate content greater than 10% may cause soils to expand more than desired [14]). According to EN 14227-4 [15], fly ash is classified into calcareous (type W, equivalent to ASTM class C), and siliceous (type V, equivalent to ASTM class F).

Class C fly ash is mainly produced from lignite or subbituminous coal. This coal has a higher content of calcium carbonate, so class C fly ash is rich in calcium (more than 20% CaO), resulting in the self-cementing characteristics. Studies concerning fly ash utilization for soil stabilization indicate that class C fly ash is an effective and economical stabilizer for broad engineering applications [14,16,17,18,19,20,21,22,23].

Class F fly ash is mainly produced from burning anthracite or bituminous coal. This class of fly ash has pozzolanic properties, but has no self-cementing characteristics due to its lower CaO content (less than 10%). According to [24], class F fly ash should be used in soil stabilization with the addition of cementitious agent (lime, lime kiln dust, cement and cement kiln dust). However, there are researches indicating that this fly ash can effectively improve some engineering properties of soil (unconfined compressive strength (UCS), California bearing ratio (CBR) and swell potential) without activators [25,26,27,28,29].

According to [14,17,30], the optimal fly ash content for soil stabilization is in the range from 10% to 30%, depending on soil and ash type. Recent studies have shown that compaction properties and the strength of the mixture decreases with the increase in compaction delay time, which is a consequence of the loss of established cement bonds between particles and lower density [14,16]. According to the same research, it is proposed to carry out compaction within two hours after mixing. Water content of the soil during compaction has a major impact on density and strength of the mixture. According to [14,16], the water content for achieving the maximum strength is typically the optimal water content or up to 8% lower than the optimal.

The effects of applying fly ash for soil stabilization are the reduction in the plasticity and soil swell potential and increasing the soil strength and CBR values. The size of fly ash particles is commonly larger than the clay particles, thus the addition of fly ash changes the grain size distribution of the clay and reduces the liquid limit. The chemical composition of ash and treated soil also affects the Atterberg limits. Reduction of plasticity of the clay soil leads to a decrease in swell potential. Çoçka [17] as well as Nalbantoglu and Gucbilmez [30] have found that plasticity and swelling potential decrease with the increase in the content of class C fly ash. Ramadas et al. [26] have analyzed the characteristics of three expansive soils with the addition of class F fly ash of 0–50%, which resulted in significant decrease in liquid limit, swelling pressure and potential. 

The increase in strength is the main reason for the use of fly ash for soil stabilization [24]. The California bearing ratio value is the primary parameter in the evaluation of suitability of fly ash stabilized soil utilization in road construction [14,16,20,22,31]. Clays generally have low CBR, and that makes them inappropriate for the use in base layers of pavements. Zia and Fox [32] have found that CBR values of loess increased five times by the addition of 10% class C fly ash. By adding 20% of self-cementing fly ash to fine-grained soil, White et al. [22] obtained CBR values that correspond to well-compacted gravel (~75%). Acosta et al. [18] investigated different soil types with very low CBR values (0–5%) and by the addition of 18% class C fly ash, achieved a significant increase in CBR values (20–56%). Vukićević et al. [29] analyzed the effect of class F fly ash on the strength of expansive clay. The highest increase in strength was obtained with the addition of 15% fly ash. The CBR value increased almost three times.

Increase of fly ash stabilized soil strength is a time-dependent process. The study of White et al. [22] on self-cementing fly ash showed a rapid increase in strength during the first 7 to 28 days, after which the slow down trend was registered due to prolonged pozzolanic reactions. 

### 2.2. Ash Utilization as a Material for Embankment

Ash has been used for many years in construction as fill material in road construction, embankment construction and land reclamation [33]. Low compacted unit weight of fly ash makes it very suitable material in embankment construction. 

Class F fly ash is more often applied as the material for embankments and backfills, in comparison with the class C fly ash [34], because of self-cementing characteristics of C class fly ash, which hardens within 2–4 h after the addition of water [35].

The important engineering properties of ash for its utilization in roads construction are: The moisture–density relationship, shear strength and compressibility. 

Fly ash has a lower compacted density compared to traditional materials, which leads to smaller applied load and settlement of the subsoil. DiGioia et al. [36] have investigated the maximum dry density and the optimum water content for Western Pennsylvania class F fly ash and Western USA class C fly ash. Values of the maximum dry density varied from 11.9 to 18.7 kN/m^3^ and values of the optimum water content from 13% to 32%. They concluded that the large variations were due to different physical and chemical characteristics of the ashes, which in the turn depend on the source of coal and the condition of coal combustion.

Shear strength tests on compacted ash specimens indicate that ash strength is mostly generated by internal friction [37]. Class F fly ash has a friction angle usually in the range of 26° to 42° [38]. Kim et al. [37] conducted tests on a mixture of fly ash and bottom ash and obtained friction angles in the range of 28–48°, which is in the rank of the shear strength of dense sandy soil.

There is not much published data for the California bearing ratio of ash. According to [39], CBR for class F fly ash ranges between 6.8% and 13.5% in the soaked conditions, and between 10.8% and 15.4% in the unsoaked conditions. For natural soils, CBR values normally range between 3% and 15% (fine-grained soils), 10–40% (sand and sandy soils) and 20–80% for gravels and gravelly soils [40].

Generally, technical standards prescribe that embankment must have small compressibility to reduce roadway settlements. The compressibility can be expressed through compression index Cc and recompression index Cr or through compressibility (constrained) modulus Mv (Cm). Kaniraj and Gayathri [41] carried out consolidation tests on the specimens of class F fly ash from the Dadri thermal power plant (New Delhi, India). They found that the compression indices Cc of the specimens were 0.041 or 0.084, depending on level of effective stress. The average recompression index Cr was 0.008. For the fly ash from the Rajghat thermal power plant (New Delhi), reported Cc and Cr were 0.072 and 0.017, respectively [42]. For the fly ashes from USA and Canada, McLaren and DiGioia [43] found the mean value of 0.13 for Cc. Kim and Prezzi [44] determined the tangent constrained modulus Cm at vertical stresses, from zero to 200 kPa, which is the range of stresses expected in highway embankments. The fly ash used in the study was class F, from three power plants in Indiana (USA). The obtained values were compared with the tangent constrained modulus available in the literature for compacted sand at different densities (at relative compaction of 99% and 85%). Specifically, the values of constrained moduli for the tested fly ashes (in the range of 10 MPa to 30 MPa at stress level 100–200 kPa) correspond to the lower end of the sand moduli range. This indicates that for the same compaction levels, the fly ashes are slightly more compressible than sand.

## 3. Materials 

### 3.1. Soil

Soil was sampled from the location Radljevo, municipality Ub, Serbia. Based on the modified Unified Soil Classification System, the tested soil was high plasticity (CH) clay. Nevertheless, due to demonstrated shortcomings of the Casagrande chart [45], as an alternative, the authors used a new classification approach proposed in [46]. Moreno-Maroto and Alonso-Azcárate [46] classified clay by PI/LL (Plasticity Index vs. Liquid Limit) ratio. The PI/LL ratio for the tested clay was 0.38, which characterized the used material as moderately or slightly clayey soil. The maximum toughness, T_max_, parameter that best represents plasticity [47], estimated by the Moreno-Maroto and Alonso-Azcárate [46] equation was 5.54, which indicates a low influence of clay minerals. Basic physical properties of CH clay are given in Table 1. The tested soil had a low to moderate swelling potential, with a swell deformation of 2.2% [48], which makes it generally unusable for most engineering purposes. 

### 3.2. Ash and Slag

In the scope of this paper, the following waste materials from Serbian power plants were used:

(1) KOL FA—fly ash from electrostatic precipitators in thermal power plant “Kolubara”;

(2) KOS FA—fly ash from electrostatic precipitators in thermal power plants “Kostolac A” and “Kostolac B”;

(3) KOS AB—ash and slag mixture from the landfills of thermal power plants “Kostolac A” and “Kostolac B”;

(4) TENT A—ash and slag mixture from the landfill of thermal power plant “Nikola Tesla A”;

(5) TENT B—fly ash from the silos in thermal power plant “Nikola Tesla B”.

Basic physical properties of tested waste materials are given in Table 2. 

According to the standard ASTM C618 [13], the used materials belonged to class F. Chemical composition of all waste materials within this paper is given in Table 3. 

### 3.3. Binders (Activators)

The influence of common hydraulic binders on soil stabilization (cement and hydrated lime) was investigated in this paper. Specifically, Portland cement PC 20M (S-L) 42.5R “Beočin Profi” with the mixed addition of granular slag and limestone from the manufacturer “Lafarge”(Beočin, Serbia) was used. Important technical specifications for used cement are given in Table 4. On the other hand, in the case of lime, hydrated lime from the “NEXE” (Jelen Do, Serbia) manufacturer was employed. Besides binders, liquid additive Polybond^TM^ from “Superroads Technologies” (Lausanne, Switzerland) was also used. Polybond^TM^ is based on sulfuric acid and surfactant, which improves soil strength and soil resistance to moisture infiltration and frost. The Polybond^TM^ stabilization effect is based on its ability to perform ionic water substitution on the soil particles’ surface using stabilizing molecules. The main feature of the stabilizing molecules attached to the soil particles’ surface is to repel moisture, thereby reducing the ability of clay particles to attract water [49]. Addition of Polybond^TM^ increases the ultimate compression strength of specimens after 28 days by 1.5–2 times compared to reference specimens [49].

## 4. Testing Methods and Laboratory Program

### 4.1. Testing Methods

Physical and mechanical properties were determined via the following laboratory tests: Specific gravity, grain size distribution, the moisture–density relationship (Proctor compaction test), unconfined compressive strength (UCS), direct shear, consolidation, CBR and swell tests. Tests were performed in accordance with Serbian (SRPS/EN) standards (see References 50–58). Additional details of laboratory tests are as follows:

- Specific gravity was determined in accordance with [50].
- Grain size distribution was determined using the hydrometer method, in accordance with [51].
- Аtterberg limits were determined using a motorized Casagrande liquid limit device (Controls, Milan, Italy), in accordance with [52].
- The Proctor compaction test was done in accordance with [53]. Optimum moisture content (OMC) and maximum dry density γ_d,max_ were determined using a compaction energy of 600 kJ/m^3^.
- Unconfined compression (UCS) tests were done using a controlled strain rate machine (Controls, Milan, Italy), on the cylindrical specimens with a diameter of 38 mm and height of 76 mm. The tests were done in accordance with SRPS U.B1.029:1996 [54]. The rate of vertical displacement was 0.5 mm/min.
- Direct shear tests were performed in drained conditions, using machines with a constant strain rate and square shear box (60 mm × 60 mm × 30 mm), in accordance with [55]. Specimens were initially saturated in a separate consolidation device (Controls, Milan, Italy) during 24 hours. After saturation, specimens were consolidated with vertical loading of 100, 200 and 400 kPa and then sheared with the constant velocity of 5–15 μ/min (CH clay stabilization) and of 20–40 μ/min (fly ash and ash-slag mixtures).
- One-dimensional consolidation tests were done in accordance with [56], on cylindrical specimens with diameter of 70 mm and height of 20 mm. The specimens were soaked for 24 hours prior to compression. After soaking, the vertical load was applied step by step to achieve the maximum vertical stress of 400 kPa, according to the following scheme: 25/50/100/200/400/200/100/50/25 kPa
- California bearing ratio (CBR) tests were done on fully soaked samples, in accordance with [57].
- Frost resistance tests were done in accordance with [58]. After 15 cycles of freezing and thawing, the UCS reduction was measured. One cycle consisted of 16 hours freezing on temperature −10 °C and 8 hours thawing on temperature +25 °C.
- Free swell tests were performed in the oedometer apparatus (Controls, Milan, Italy) on remolded samples compacted at standard Proctor’s maximum dry density and optimum moisture content and without any vertical surcharge load [48]. Upon completion of the swelling process, in order to capture the swelling pressure, the vertical load was gradually applied until swelling deformation was eliminated.

### 4.2. Laboratory Testing Program

**Figure 1:**
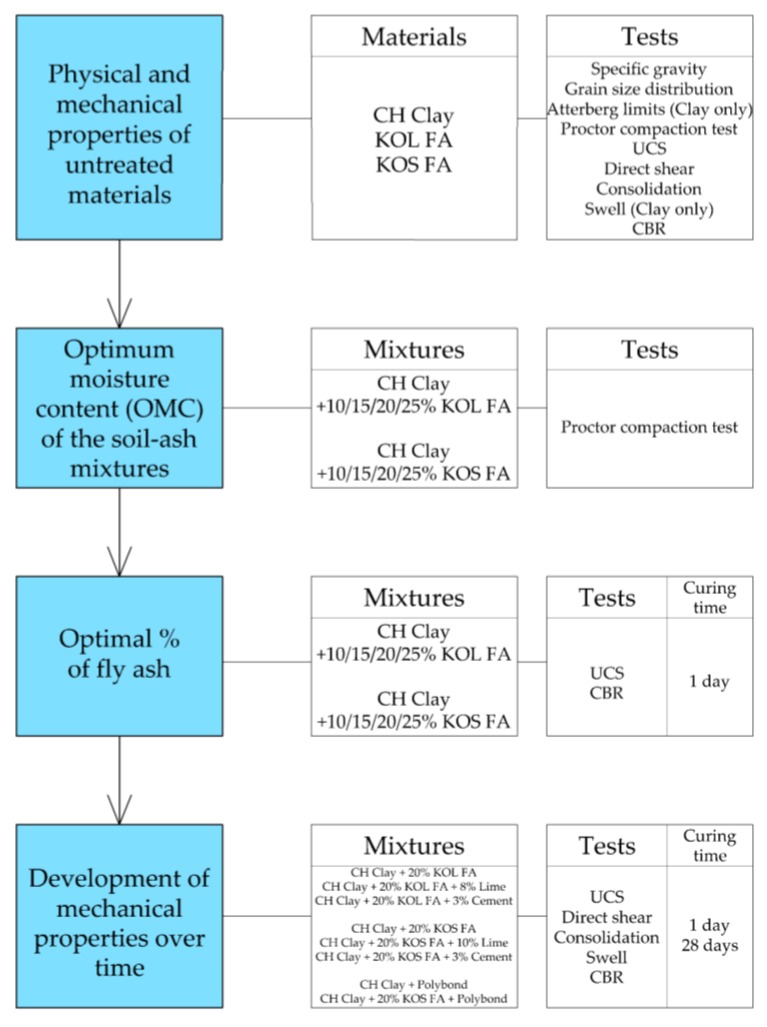
Flowchart of the laboratory testing program—high plasticity clay stabilization.

**Figure 2:**
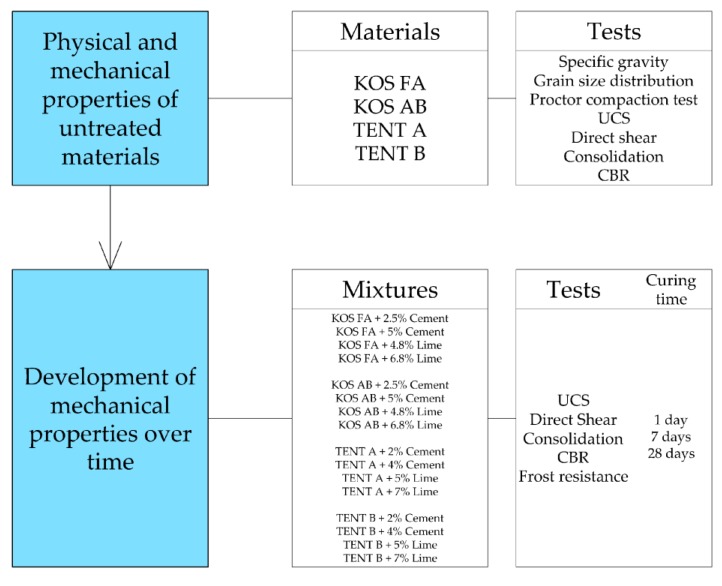
Flowchart of the laboratory testing program—ash and ash-slag mixtures.

The laboratory testing program within this study consisted of two parts. In the first part, the high plasticity clay stabilization using fly ash was investigated. In the second part, engineering properties of ash and ash-slag mixtures as an embankment material in road construction were studied. The influence of common binders (activators) was also investigated. The laboratory testing program is outlined in flowcharts in Figure 1 and Figure 2.

A total of 24 combinations (mixtures) of soil, waste material and binders (activators) were tested. Untreated materials (without binders) were tested first, in order to determine initial physical (Table 1 and Table 2) and mechanical properties (Table 5 and Table 6), which were used later for comparison with treated materials. For all physical and mechanical tests two specimens were used for the determination of engineering properties, except for UCS where five specimens were tested.

### 4.3. Specimen Preparation and Curing 

In order to compare the results of different test mixtures, specimens for mechanical tests (UCS, direct shear, CBR, consolidation and swell) were prepared by compaction under the same conditions. First, premeasured amount of dried components (ash, slag, soil and binder) were mixed intensively to create a homogeneous dry mixture. After that the water was added and, after mixing, compaction was done immediately. Late compaction can reduce the effects of stabilization—during the hydration process, fly ash cements particles in the mixture, and more compaction effort is required. The smaller strength gain, and sometimes strength reduction after late compaction, is explained by the loss of hydration products, and by the loss of connections between the cemented particles [14,59]. According to [14,60], it is recommended that the amount of added water should be about 80–110% of OMC. In this study, the 100% of OMC was adopted for specimen preparation. After compaction, the specimens were extruded from compaction molds.

Specimens without binders and specimens with cement were kept (cured) in a plastic wrap, hermetically sealed, at laboratory temperature of 20 °C. Specimens with lime and Polybond^TM^ were not hermetically closed before testing. Specimens were cured in moist chamber at relative humidity RH > 95% and laboratory temperature of 20 °C.

### 4.4. Optimal % of Fly Ash (Only for Soil Stabilization)

For the successful soil stabilization it is important to use the optimal fly ash content, in order to create the conditions necessary for all chemical reactions and for changing the soil microstructure. 

In order to determine the optimal content of fly ash, UCS tests were done on the specimens with different fly ash–soil ratios (10%, 15%, 20% and 25%), one day after compaction. Due to the fact that the increase of UCS was not significant (may be in the domain of scattering of the results), it was not possible to select the optimal % of fly ash. Therefore, additional CBR tests were performed on the specimens with the same fly ash–soil ratios. The highest CBR value was achieved for the mixture with 20% of fly ash, which was adopted as the optimal amount.

### 4.5. Used % of Binders

Used amount of cement was determined preserving the homogeneity of the mixture with minimum cement consumption.

Used amount of lime was set from the condition that the pH value of mixture shall be 12.4, securing the optimal conditions for the hydration process [61].

Test specimens with Polybond^TM^ were prepared with the minimum recommended Polybond^TM^ content according to organization standard [62] and the manufacturers’ recommendations [49]. First the Polybond^TM^ solution with water was formed, which is then added into the dry material before compaction. During preparation of the soil–fly ash mixtures, the previously determined optimal ash percentage (20%) was used. Used amounts of binders are given in Figure 1 and Figure 2.

## 5. Results and Discussion

### 5.1. Stabilization of High Plasticity (CH) Clay 

The results of the CH clay stabilization using fly ash and binders are given in following subsections. Engineering properties of stabilized mixtures were compared with the properties of untreated soil. Mechanical properties of tested materials without binders are shown in Table 5. 

For mixtures with lime, a significant increase in shear strength, compressibility parameters (constrained modulus M_v_) and CBR was obtained after one day and therefore no further testing was performed after 28 days.

#### 5.1.1. Unconfined Compressive Strength (UCS)

**Figure 3:**
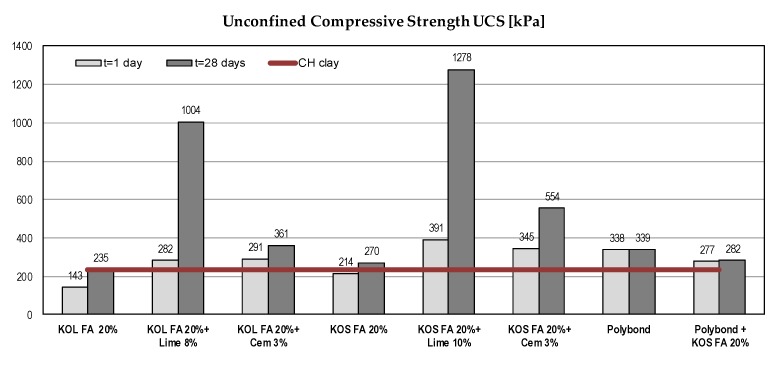
Unconfined compressive strength (UCS) of different mixtures with CH clay.

Increased soil strength is the main indicator of successful soil stabilization [14,16,18,23,31]. The results of UCS tests are presented in Figure 3. For mixtures with fly ash without a binder, the effects of stabilization were negligible because of low UCS of used fly ashes. With the addition of lime or cement, the pozzolanic reaction started and there was significant strength gain over time. Strength gain was more pronounced with the addition of lime.

The addition of Polybond^TM^ in the minimum recommended amount led to an increase of UCS after one day, but results indicate that there was insensitivity of UCS to the elapsed time. Since the Polybond^TM^ stabilization mechanism is primarily based on the reduction of bound water, the observed trend was expected.

#### 5.1.2. Shear Strength Parameters in Terms of Effective Stresses

**Figure 4:**
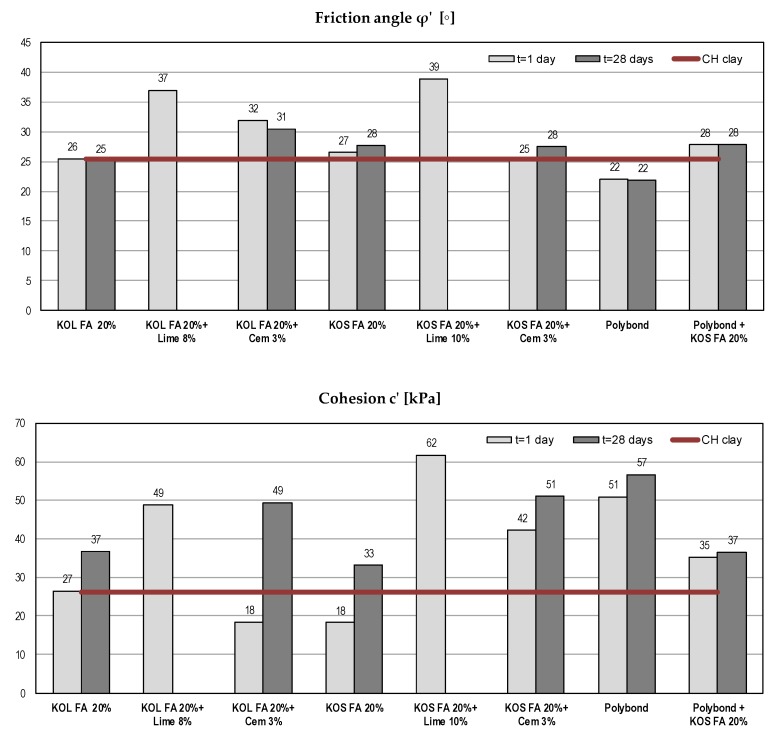
Shear strength parameters of different mixtures with CH clay.

Shear strength parameters affect the safety of any geotechnical structure. They are essential for earth structures design, calculation of bearing capacity and earth pressures, stability analysis of natural slopes, cuts and fills [63,64]. Effective shear strength parameters were determined using a direct shear test and they are presented in Figure 4.

Obtained results show that the friction angle does not substantially change with the addition of fly ash and Polybond^TM^. With the addition of cement, there was a mild increase of the friction angle, but a significant increase was noted with the addition of lime after only one day. The cohesion significantly increased with time for all tested mixtures. For mixtures with fly ash without binders, a slow pozzolanic reaction occurred due to the presence of reactive CaO. After the addition of cement or lime, a more pronounced pozzolanic reaction occurred as well as the creation of cement joints. The effects of treating CH clay with Polybond^TM^ were particularly expressed in terms of soil cohesion—the increase of cohesion was evident.

#### 5.1.3. Compressibility Parameters

**Figure 5:**
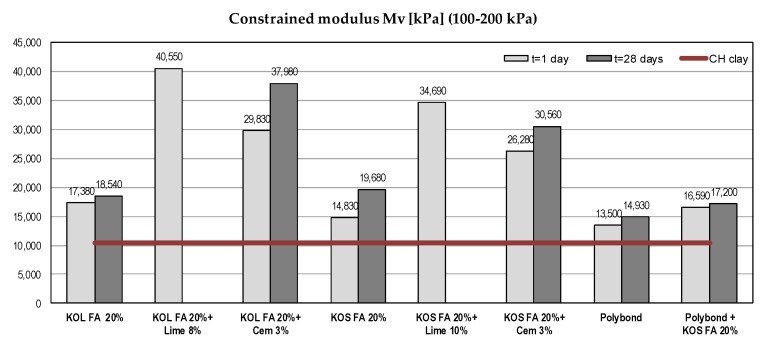
Compressibility parameters of different mixtures with CH clay.

In order to calculate the consolidation settlement of soils, compressibility parameters are required. The constrained modulus from a one-dimensional compression (oedometer) test is a commonly used parameter to determine the settlement of a tested material. The vertical effective stress level 100–200 kPa was selected to display the results. A similar trend was observed for other stress levels. Constrained moduli were increased for all mixtures (Figure 5). Stabilization effects were greater with the addition of cement or lime.

#### 5.1.4. California Bearing Ratio (CBR)

**Figure 6:**
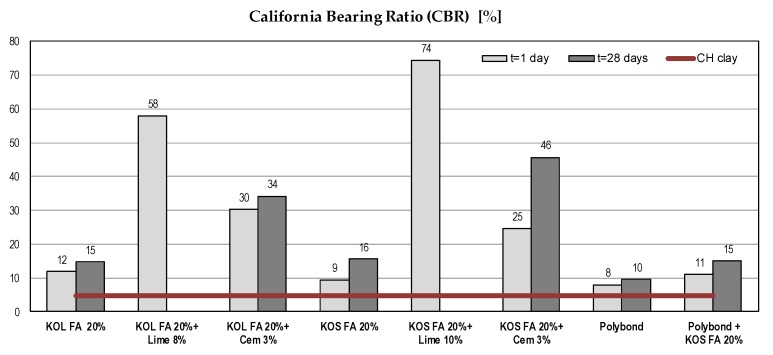
The California bearing ratio of different mixtures with CH clay.

The California bearing ratio (CBR) is a parameter that describes the strength of roads subgrade. It is used for the determination of pavement thickness and its component layers [65,66]. Clays generally have low CBR values (˂5), which make them inappropriate for road subgrade construction. Obtained results showed significant CBR gain. In the case of mixtures with fly ash and Polybond^TM^, there was a mild, but important increase of strength, which made the tested soil usable for road construction. Test results (Figure 6) were in line with [18,19,22,23,31]. It is obvious that used binders had a significant influence on CBR gain.

#### 5.1.5. Swell Potential

The volumetric change of soil causes movements in structures and imposes additional loads to structures [67,68]. According to [16], fly ash replaces some of the volume held by clay particles and acts as a mechanical stabilizer.

By addition of fly ash, the swell potential of all tested mixtures was entirely eliminated, which was somewhat expected considering the medium degree of expansivity of tested CH clay [48]. On the other hand, the addition of Polybond^TM^, reduced the swelling deformation to about 1%.

### 5.2. Fly Ash and Ash-Slag Mixtures as a Material for Embankment

The engineering properties of ash and ash-slag mixture were discussed below. As in the case of high plasticity clay, tests were performed on the samples with and without binders and the results were compared. For all tested materials similar trends were observed and therefore test results for fly ashes and ash-slag mixtures would be considered together. Mechanical properties of tested materials without binders are given in Table 6.

#### 5.2.1. Unconfined Compressive Strength (UCS)

**Figure 7:**
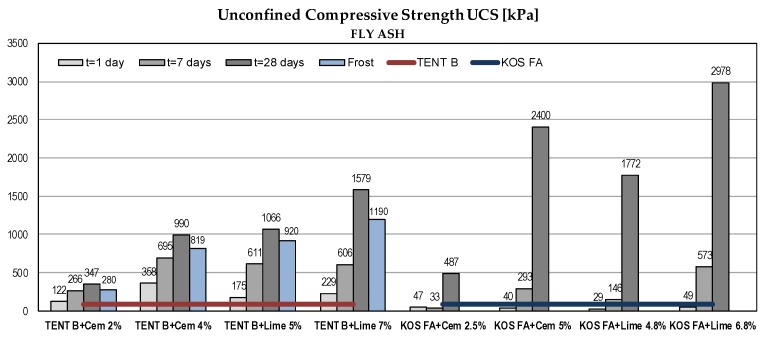

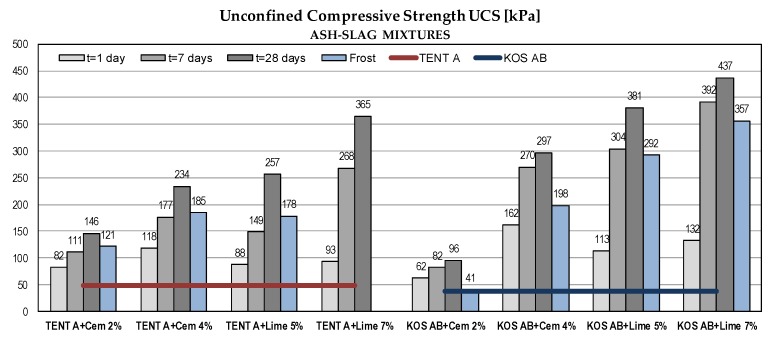
UCS of waste materials with binders.

The results of UCS tests are presented in Figure 7. Samples without a binder had very low UCS (Table 6). The pozzolanic reaction started with the addition of binders and water and constant strength gain over time could be observed. The substantial increase was recorded for fly ash samples. The strength gain was more pronounced with lime addition.

#### 5.2.2. Effect of Frost

The frost resistance of fly ash and ash-slag mixtures treated with binders was tested by measuring the UCS reduction. Samples aged 28 days were exposed to repeated freezing and thawing (15 cycles) and the UCS was determined. The frost resistance index is represented by the relation between the UCS of the sample after 15 freezing/thawing cycles and UCS of the reference sample (28 days old). According to standard SRPS U.B1.050:1970 [58], the mixture is frost resistant if the index is greater than 80%.

Results for ash-slag mixture TENT A with 7% of lime were missing because the samples were damaged during testing. Tests were not performed for fly ash KOS FA. The results are shown in Figure 7. The obtained frost resistance indices were within: 75–86% for TENT B, 69–83% for TENT A and 43–82% for KOS AB. Absolute values of UCS after freezing/thawing cycles classify mixtures as stiff to hard [64] and despite some lower indices values, the mixtures could be considered as frost resistant. The low index value for KOS AB with 2% of cement might be due to damage of samples during testing.

#### 5.2.3. Shear Strength Parameters in Terms of Effective Stresses

**Figure 8:**
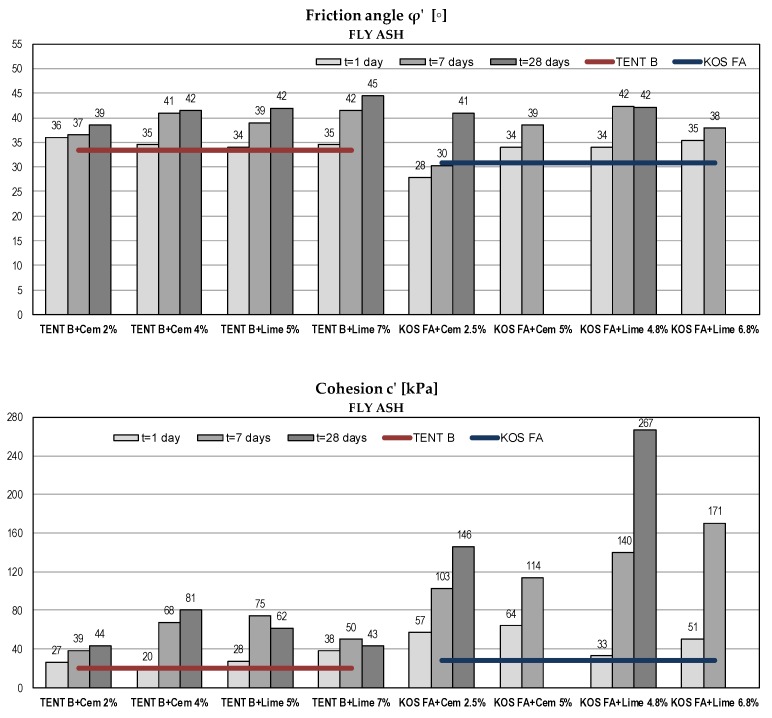
Shear strength parameters of fly ash samples with binders.

**Figure 9:**
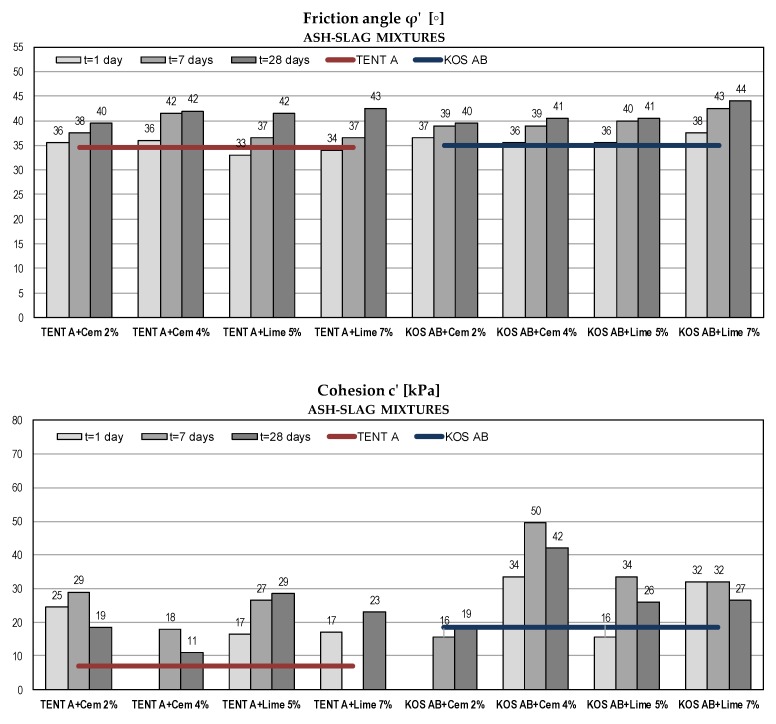
Shear strength parameters of ash-slag samples with binders (some test results were omitted because obtained values were too high).

Considering fly ash and ash-slag mixtures as a fill material for embankments, the strength of compacted material is of major importance to geotechnical engineers. High shear strength ensures higher bearing capacity and slope stability. Shear strength parameters are given in Figure 8 and Figure 9. 

Compared to the strength of the compacted sand (as traditional fill material) [37], all tested mixtures without binders had high values of friction angle (φ’ = 31–35°). According to USA Navy [69] the friction angle for compacted sandy soils typically range from 31° to 45°. Test results show an increase of shearing resistance for all samples over time and with the increase of binder amount. Similar results were obtained for both binders. Obtained friction angles after 28 days were within the range 39° to 45°, which made tested materials generally comparable to the traditional compacted sandy soils.

Considering cohesion as an apparent shear strength parameter that captures the effects of intermolecular forces, soil tension or cementation, class F fly ash and ash-slag mixtures exhibit no cohesive characteristic in the saturated state [44]. In this case, cohesion is a consequence of the approximation of the non-linear failure envelope with a linear one. The failure envelope obtained from the strength test is a curved line for mostly granular materials, but solving the majority of soil mechanics problems, it is sufficient to approximate the shear stresses as a linear function of the normal stresses. The magnitude of cohesion is thus defined by the intersect segment on the shear stress axis. After adding binders and with the addition of water, the pozzolanic reaction occurred as well as the formation of bonds between the soil particles. The increase in cohesion was evident due to the cementation process, but there was no clear trend over time and with an increase in the % of the binder. For fly ash samples, the substantial increase over time had recorded for KOS FA and for TENT B with cement addition, while with the addition of lime there was no further increase of cohesion after 7 days. For ash-slag mixtures, there was a scattering of cohesion results, probably due to the inhomogeneity of the samples and due to the method used for strength determination. In the direct shear test, the orientation of the failure plane was predetermined as being near the middle of the sample height. Better results might be obtained in the triaxial device where the orientation of the failure plane is governed by the soil structure.

For fly ash samples KOS FA with higher % of binders (5% cement and 6.8% lime), no further testing was carried out after 28 days, because there was a significant increase in tested parameters for a smaller binder amount. Some test results for ash-slag mixtures were omitted because the obtained values were too high.

#### 5.2.4. Compressibility Parameters

**Figure 10:**
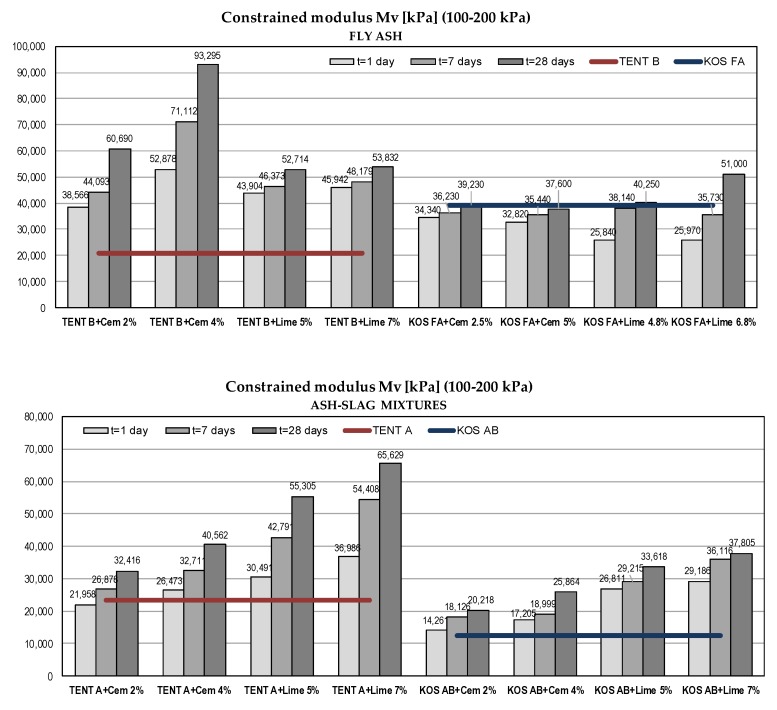
Compressibility parameters of waste materials with binders.

Compression of compacted fly ash or ash-slag mixture in wide embankments can be considered as one dimensional [37]. Thus, constrained moduli from oedometer (one-dimensional consolidation) tests were obtained and results are shown in Figure 10. According to Kim et al. [37] and Carrier [70] relevant constrained moduli should be calculated for vertical stresses ranging from zero to 200 kPa, a range of stress levels typically expected in highway embankments.

Ash-slag samples without binders exhibited slightly greater compressibility than fly ash samples. With the addition of binders constrained moduli of fly ash TENT B and ash-slag mixtures increased with time and with the percent of the binder. For fly ash samples KOS FA the stabilization effects were negligible. 

**Figure 11:**
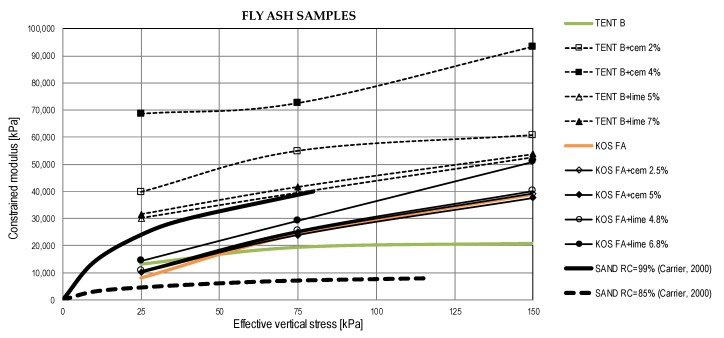

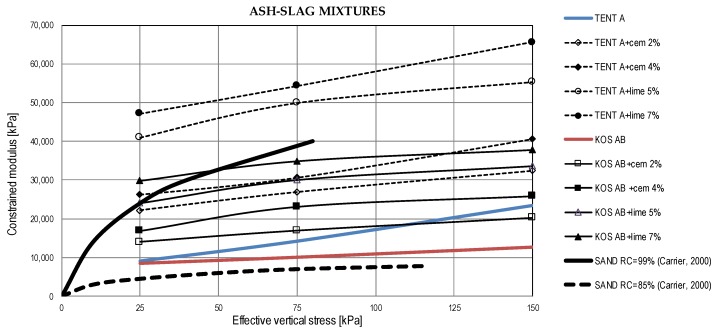
Constrained moduli of waste materials and sands.

Additionally, a comparison was made with sand compressibility given in [70]. Figure 11 shows typical moduli values for sand compacted at relative compaction (RC) of 85% and 99% and moduli (after 28 days) obtained from research as a function of vertical effective stresses. Constrained moduli are shown for the midpoint of the stress interval for which they are calculated. For the same compaction levels, fly ash samples TENT B with binders and ash-slag mixtures TENT A with lime are significantly less compressible than sand, while most of the other values of moduli lie near the upper limit of the sand moduli range.

#### 5.2.5. California Bearing Ratio (CBR)

**Figure 12:**
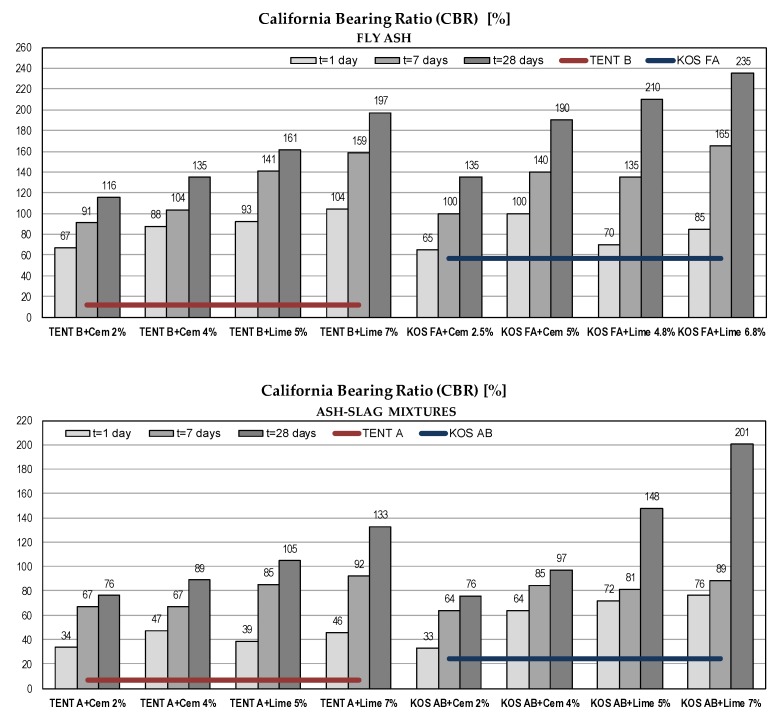
The California bearing ratio of waste materials with binders.

CBR values of tested materials vary within wide limits, from 7% for ash-slag mixture TENT A to 57% for fly ash KOS FA. With the use of binders, for all tested samples, there is a clear trend of CBR increase over time (Figure 12). Except for ash-slag mixture TENT A, there is no need for stabilization of tested materials for road subgrade construction purposes [40].

## 6. Conclusions

Thermal power plants have multiple negative effects on the environment: They pollute air with harmful gases and fly ash; landfills of ash and slag occupy large areas of mainly agriculture land; deposited ash can potentially pollute land and water due to the presence of trace elements and radionuclides. The amount of deposited ash and slag can be significantly reduced by use in the construction industry.

In order to assess the applicability of fly ash and ash-slag mixtures for subsoil stabilization and embankments construction, the laboratory tests on different mixtures of soil, ash, slag and binders were performed. The soil stabilization efficiency of a non-self-cementing class F fly ash without a binder was tested, as well as the effects of adding a binder as the cementation agent. The characteristics of fly ashes and ash-slag mixtures as the construction material were also investigated.

Considering the results of CH clay stabilization, fly ashes from power plants Kolubara and Kostolac could be successfully used as an additive that improves all mechanical characteristics of the soil required for the subsoil. Due to improved mechanical parameters, the stabilized soil has better bearing capacity and low compressibility. The increase in CBR values and elimination of swell potential make tested soil usable for road construction. With the addition of binders, all tested engineering properties were significantly improved. The addition of lime yields more significant stabilization results compared to cement.

Regarding the embankments and road subgrade design purposes, fly ash and ash-slag mixtures from Serbian thermal power plants have comparable mechanical properties with sands. The use of binder contributes to the substantial increase of shear strength parameters, compressibility modulus and stiffness of tested materials. Achieved high shear strength of waste materials ensures higher bearing capacity and slope stability. Low compressibility also makes waste materials suitable for embankment construction. The use of ashes and ash-slag mixtures as construction material provides multiple benefits: Reduced amount of ash on landfills, preservation of natural resources, lower price of embankment construction, lower energy consumption and CO_2_ emission.

## Figures and Tables

**Table 1:** Physical properties of the high plasticity (CH) clay.

G_s_	Grain Size Distribution					Atterberg Limits			Swell %
	Clay <0.002 mm	Silt 0.002–0.06 mm	Sand 0.06–2 mm	Gravel 2–60 mm	Fines <0.075 mm	LL %	PL %	PI %	
2.67	22	72	6	-	96	51.0	31.5	19.5	2.2

Note: Testing methods are described in Section 4.

**Table 2:** Physical properties of tested waste materials.

Material	G_s_	Grain Size Distribution (%)				
		Clay <0.002 mm	Silt 0.002–0.06 mm	Sand 0.06–2.0 mm	Gravel 2–60 mm	Fines <0.075 mm
KOL FA	2.13	0–2	60–65	35–38	-	67–72
KOS FA	2.22	-	75	25	-	80
KOS AB	2.41	2	10–22	77–89	-	14–27
TENT A	2.39	0–1	40–41	57–58	-	49–50
TENT B	2.26	2	14–31	65–82	2	22–40

Note: Testing methods are described in Section 4.

**Table 3:** Chemical composition of the used waste materials.

Material	Chemical Composition (%)									
	SiO_2_	Al_2_O_3_	Fe_2_O_3_	CaO	MgO	K_2_O	Na_2_O	TiO_2_	SO_3_	P_2_O_5_
KOL FA [7]	50.21	23.83	9.89	4.79	3.12	0.44	0.35	0.54	5.24	0.060
KOS FA [7]	56.38	17.57	10.39	7.46	2.13	0.57	0.38	0.52	0.95	0.025
KOS AB [6]	53.61	17.72	8.05	7.44	1.78	1.22	0.86	0.51	0.12	0.068
TENT A [6]	56.14	15.93	5.77	7.54	1.48	1.23	0.86	0.52	0.12	0.058
TENT B [6]	59.73	20.97	5.99	5.83	2.21	1.18	0.41	0.57	0.48	0.023

Note: Presented values may not entirely represent the tested material, since the chemical composition of the coal used in the power plants can change over time.

**Table 4:** Technical properties for cement PC 20M (S-L) 42.5R.

Consistency (%)	Setting Time (min)	Compressive Strength After 2 Days (MPa)	Compressive Strength After 28 Day (MPa)
27–29	160–250	26–28	49.5–54.5

**Table 5:** Mechanical properties of tested materials without binders.

Material	Compaction		Compressibility			Strength			
	Proctor Test (600 kJ/m^3^)		M_v_ (kPa)			Direct shear		UCS	
	OMC (%)	γ_d,max_ (kN/m^3^)	50–100 kPa	100–200 kPa	200–400 kPa	φ’ (°)	c’ (kN/m^2^)	q_u_ (kN/m^2^)	CBR (%)
CH clay	19.1	16.6	14300	10400	10800	25.5	26.0	231	4.5
KOL FA	49.8–55.0	8.0	17700	24900	31400	29.5	36.5	83	13
KOS FA	37.5–43.9	9.0–9.8	25800	39200	42900	31.0	28.5	87	57

**Table 6:** Mechanical properties of tested materials without binders.

Material	Compaction		Compressibility			Strength			
	Proctor Test (600 kJ/m^3^)		M_v_ (kPa)			Direct Shear		UCS	
	OMC (%)	γ_d,max_ (kN/m^3^)	50–100 kPa	100–200 kPa	200–400 kPa	φ’ (°)	c’ (kN/m^2^)	q_u_ (kN/m^2^)	CBR (%)
KOS FA	37.5–43.9	9.0–9.8	25800	39200	42900	31.0	28.5	87	57
KOS AB	48.1	9.1	10000	12600	22700	35.0	18.5	37	24
TENT A	48.5	8.9	14100	23300	34200	34.5	7.0	49	7
TENT B	33.7	10.4	19300	20700	26200	33.5	20.0	87	12
